# Karyotype diversity between species of *Crenicichla* (Perciformes, Cichlidae) from different Brazilian hydrographic basins

**DOI:** 10.1590/1678-4685-GMB-2018-0052

**Published:** 2019-02-18

**Authors:** Larissa Bettin Pires, Mariana Campaner Usso, Lucia Giuliano-Caetano, Ana Lúcia Dias

**Affiliations:** 1 Departamento de Biologia Geral, Centro de Ciências Biológicas, Universidade Estadual de Londrina, Londrina, PR, Brazil

**Keywords:** Chromosome banding, fish cytogenetics, Geophaginae, ribosomal DNA

## Abstract

*Crenicichla* is the largest genus in the Cichlidae family in South America. The genus includes 100 valid species that are popularly known in Brazil as *jacundás* or *joaninhas* and are widely distributed in rivers east of the Andes. Cytogenetic analyses were carried out on seven species in this genus*.* All species showed a diploid number of 48 with interspecific differences in karyotype formulas and AgNORs located in interstitial position on the short arm of the largest metacentric pair, except for the two populations from *C. britskii.* Population A showed terminal markings on the long arm of the fifth pair of the complement, and population B showed up to two marked chromosome pairs. FISH with an 18S rDNA probe was coincident with AgNORs and CMA_3_, except for pair 6 from population B of *C. britskii* that did not presented positive CMA_3_ sites. This work presents first cytogenetic data for *C. haroldoi*, *C. maculata,* and *C. punctata*, and the results show karyotypic patterns similar to those in the literature. However, the diversity found in populations of *C. britskii* represents new information about the evolution of the karyotype of the Cichlidae family, which has been conservative. Furthermore, the data could assist in phylogenetic studies of *Crenicichla*.

## Introduction

The Cichlidae family includes a wide variety of fish species and is one of the largest in Perciformes. There are approximately 1706 valid species (Eschmeyer and Fong, 2018), and the group is considered highly specialized (Kullander, 1998). Through cladistics morphological analyses, Kullander (1998) verified that this family is a monophyletic group and showed a dichotomy between “Old World” and “New World” cichlids.

Stiassny (1991) first recognized the monophyletism of Neotropical cichlids, which include more than 406 valid species (Kullander, 2003). This was later confirmed by phylogenetic relationships based on molecular data (Farias *et al.*, 1999; López-Fernández *et al.*, 2010) and combinations of morphological and molecular data (Farias *et al.*, 2000; López-Fernández *et al.*, 2005; Smith *et al.*, 2008). Among Neotropical cichlids, the genus *Crenicichla* is one of the most numerous, with 100 valid species described (Frose and Pauly, 2018). The pike cichlids are easily recognized by their elongated body, large mouth, and prognata. These cichlids mostly occur in tropical and subtropical regions of South America, from the coastal drainages of Venezuela and Guiana to the Plata River in Argentina (Kullander and Lucena, 2006).

This genus has been studied extensively from a cytogenetic point of view, with the first work conducted by Oyhenart-Perera *et al.* (1975) on *Crenicichla sexatilis*. Since then, several studies have been carried out, and the majority identify only the diploid number (2n), with a total of 19 species analyzed to date presenting a conserved 2n equal to 48, according to cytogenetic surveys performed by Feldberg *et al.* (2003) and Benzaquem *et al.* (2008). Only *Crenicichla* sp. does not present 48 chromosomes, showing 2n=46 (Rezende *et al.*, 1996). The phylogenetic position of *Crenicichla* within the family is quite controversial, sometimes being assigned to the clade Cichlinae (Stiassny, 1991; Kullander, 1998) and sometimes to the clade Geophaginae (Farias *et al.*, 2000; López-Fernández *et al.*, 2005; Landim, 2006; Smith *et al.*, 2008).

Thus, the aim of this work was to perform conventional and molecular cytogenetic analyses of seven pike cichlids species: *Crenicichla britskii, C. lepidota, C. niederleinii, C. semifasciata, C. punctata, C. haroldoi,* and *C. maculata.* The results provide the first karyotypic information for the last three species. The data presented could be used as an additional tool for phylogenetic studies and help to better define relations within the genus, as well as improve the understanding of the karyotype evolution of the group.

## Materials and Methods

The seven species studied were collected from four Brazilian hydrographic basins (Table 1).The specimens were deposited in the Museum of Zoology at the State University of Londrina, Parana, Brazil. For convenience, different populations of *C. britskii* were called population A (Taquari) and population B (Paranapanema), as shown in Table 1.

**Table 1:** Collection sites and hydrographic basins of *Crenicichla* specimens analyzed. MS = Mato Grosso do Sul; PR = Paraná; RS=Rio Grande do Sul

Species	Collection sites	Hydrographic basins	Number of individuals
*Crenicichla britskii*	Taquari stream-PR (A) 23º10’45.2’S 50º56’30.9’’W	Paranapanema river	7M,6F
	Paranapanema-SP (B) 22º42’30.3’’S 51º04’08.4’’W		
*C. haroldoi*	Pavão stream / PR	Paranapanema river	2M,2F
*C. niederleinii*	Três Bocas stream- PR 23º23’06.6 ‘S 51º04’35.8 ‘’ W	Paranapanema river	2M,5F
*C. lepidota*	Miranda river-MS 19º34’38.01 ‘S 57º01’06.63’W	Paraguai river	1M,2F
*C. semifasciata*	Miranda river-MS 19º34’38.01 ‘S 57º01’06.63’W	Paraguai river	1F
*C. maculata*	Maquiné river-RS 29º39’10.4 ‘S 50º12’31.8’’W	Tramandaí river	2M,4F
*C. lepidota*	Barra do João Pedro-RS 29º46’21.2 ‘S 50º05’08.0’’W	Tramandaí river	3M,3F,3?
*C. punctata*	Saco da Alemoa and river Forqueta-RS 29º22’08.0 ‘S 52º03’30.0’’W	Laguna dos Patos System	2M,5F
	**Total of individuals: 50**		

M: male. F: female.

Mitosis was stimulated by the injection of yeast suspension in animals, as described by Lee and Elder (1980). Mitotic chromosomes were obtained by direct preparation by removing the anterior kidney according to the methodology proposed by Bertollo *et al.* (1978), and slides for conventional analysis were stained with 5% Giemsa stain in phosphate buffer at pH 6.8. The morphology of the chromosomes was determined based on the ratio of arms, as proposed by Levan *et al.* (1964). For determination of the fundamental number (FN), the metacentric (m) and submetacentric (sm) chromosomes were considered biarmed and the subtelo-acrocentric (st-a) uniarmed.

Nucleolar organizer regions (NORs) were detected by impregnation with silver nitrate according to the technique described by Howell and Black (1980). GC- and AT-rich sites were detected with chromomycin A_3_ (CMA_3_) and 4’, 6-diamino-2-phenylindole (DAPI) according to Schweizer (1980). Fluorescence *in situ* hybridization (FISH) was performed according to the protocol from Pinkel *et al.* (1986) with modifications according Gouveia *et al.* (2013) using a 18S rDNA probe (Hatanaka and Galetti Jr, 2004). Finally, the slides were analyzed on an epifluorescence microscope (Leica DM2000), which was attached to a digital camera. Metaphase images were captured using Leica Application Suite version 3.1.0. (Leica Microsystems).

## Results

**Figure 1:**
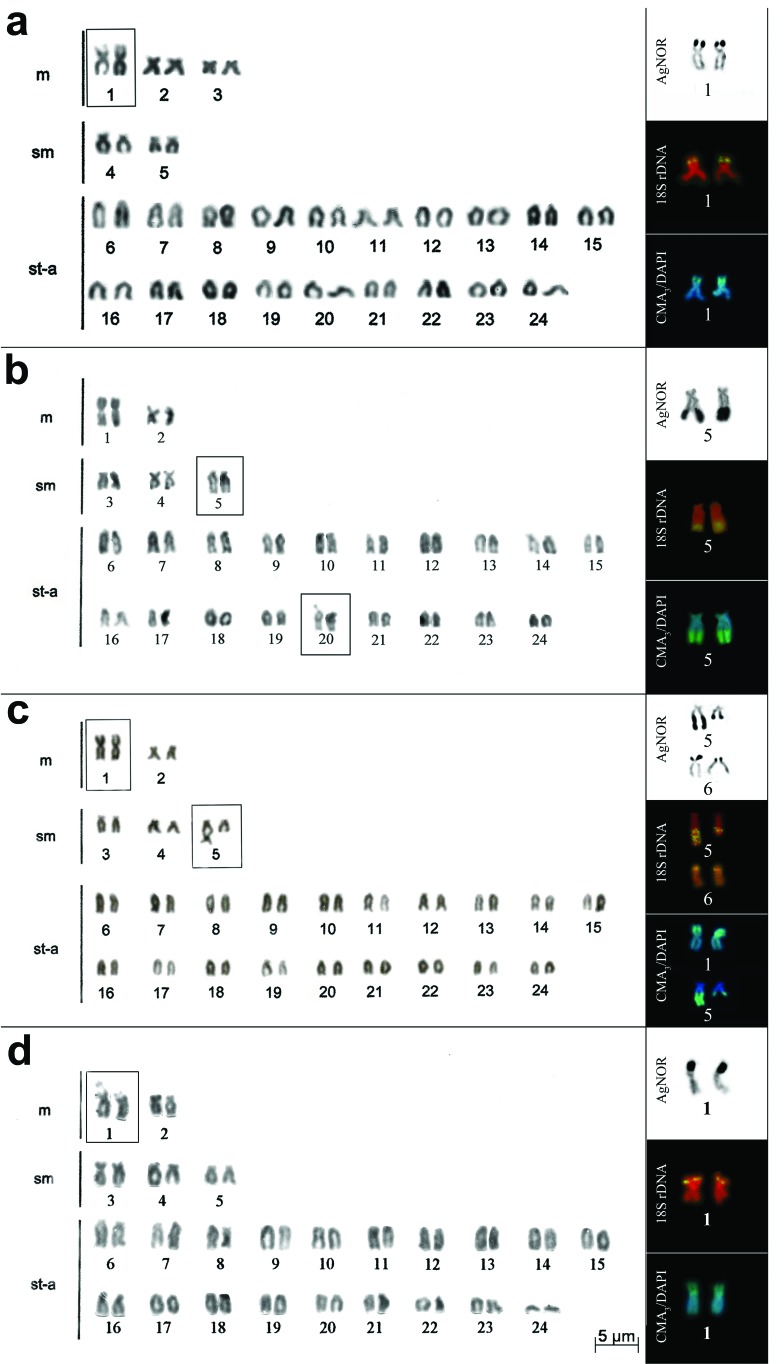
Karyotype and chromosome pairs with silver nitrate staining, FISH with 18S rDNAr probe and CMA_3_/DAPI in: *Crenicichla haroldoi* (a), *C. britskii*, populations A (b) and B (c), and *C. niederleinii* (d), respectively. In the boxes are secondary interstitial constrictions in the short arm of the first metacentric pair (a, d) and in the long arm of the fifth pair (b, c).

**Figure 2:**
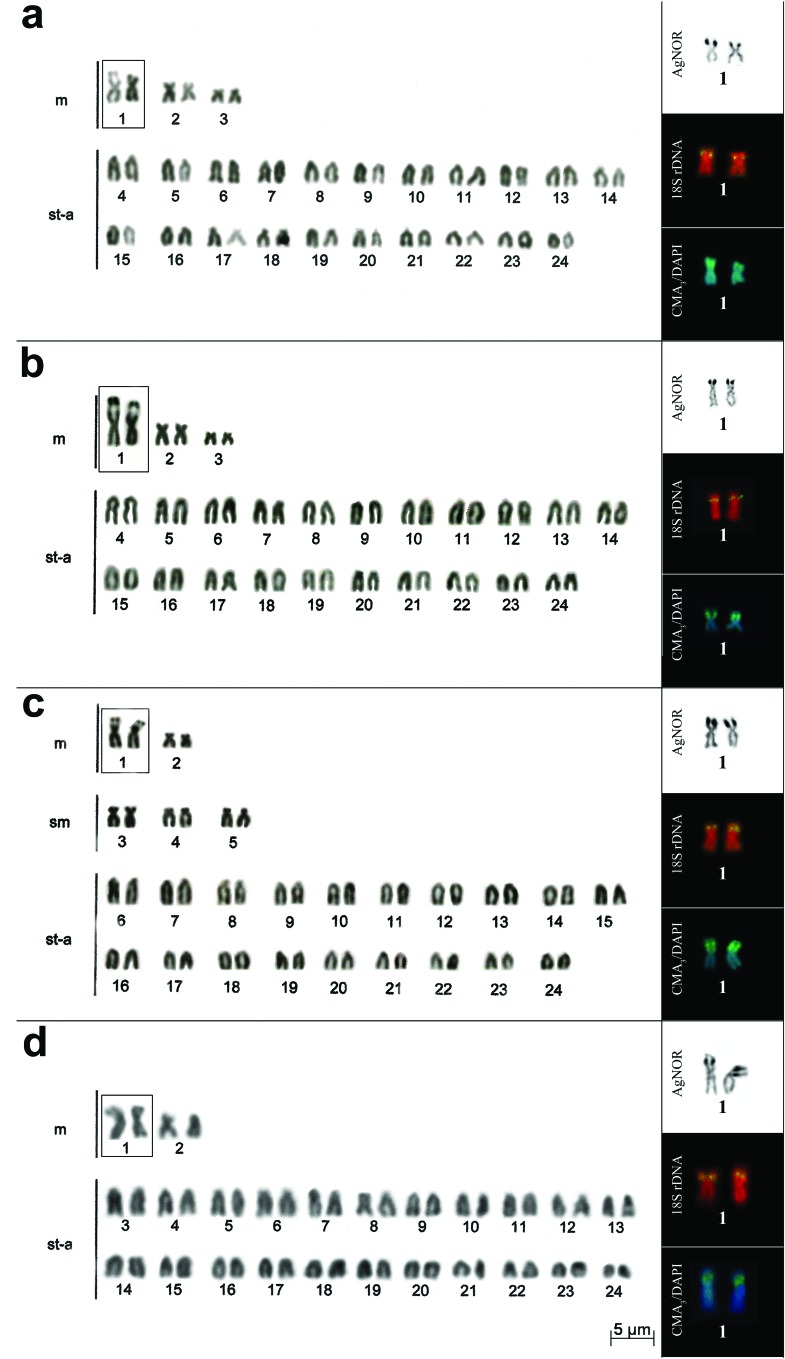
Karyotype and chromosome pairs with silver nitrate staining, FISH with 18S rDNAr probe and CMA_3_/DAPI in: *Crenicichla maculata* (a), *C. lepidota* (b), *C. punctata* (c) and *C. semifasciata* (d), respectively. In the boxes are secondary interstitial constrictions in the short arm of the first metacentric pair.

All species analyzed showed a diploid number (2n) of 48 chromosomes, but four different karyotype formulas among species were observed: 6m+4sm+38st-a and FN=58 for *C. haroldoi* (Figure 1a), 4m+6sm+38st-a and FN=58 for *C. britskii*, *C. niederleinii,* and *C. punctata* (Figure 1b-d and Figure 2c), 6m+42st-a and FN=54 for *C. maculata* and *C. lepidota* (Figure 2a,b), and 4m+44st-a and FN= 52 for *C. semifasciata* (Figure 2d).

AgNORs were located on a pair of chromosomes for all species (Figure 1a,b,d and Figure 2a-d), except for population B from *C. britskii,* which showed up to two marked chromosome pairs (Figure 1c). Population A of *C. britskii* showed terminal markings on the long arm of the fifth pair of the complement (sm) (Figure 1b). All other species showed NORs in an interstitial location on the short arm of the largest metacentric pair (boxes in Figure 1a,d and Figure 2a-d).

The AgNORs were coincident with the secondary constrictions observed by Giemsa staining. Exceptions were observed in *C. britskii*. In population A, the secondary constriction observed in pair 20 was not a positive AgNOR, only the constriction of pair 5 (Figure 1b, box). In population B, pair 5 showed a heteromorphism of NORs in the long arm coincident with the secondary constriction, and pair 6 showed a heteromorphism of NORs in the short arm that was not coincident with secondary constriction (Figure 1c, box).

For all species of *Crenicichla* the FISH analysis with the 18S rDNA probe was coincident with AgNORs (Figures 1 and 2).

Staining with CMA_3_ showed fluorescent markings coinciding with the NORs in all species analyzed (Figures 1 and 2), except pair 6 from population B of *C. britskii*. In this population, there was an additional positive CMA_3_ pair (pair 1), as shown in Figure 1c. Size heteromorphism with CMA_3_ occurred in pair 5 of *C. britskii* from population B and in pair 1 of *C. niederleinii* and *C. maculata, as* evidenced by Giemsa staining and with the 18S rDNA probe (Figure 1c,d, Figure 2a, Table 2). In DAPI staining, the NORs did not showed fluorescent signals, appearing only as a negative band (Figures 1 and 2).

**Table 2:** Karyotype results for the *Crenicichla* species analyzed in the present study: 2n = diploid number, FN = fundamental number, SC = secondary constriction, NORs = nucleolar organizer regions and CMA_3_ = chromomycin A_3_, t=terminal, i=interstitial, * heteromorphism.

Species	Locality	Populations	2n	Karyotypic formula	FN	SC	NORs	CMA_3_
*C. britskii*	Taquari stream (PR)	**A**	48	4 m + 6 sm + 38 st-a	58	Pair 5 (t) Pair 20 (t)	Simple: Pair 5 (t)	Pair 5 (t)
	Paranapanema river (SP)	**B**	48	4 m + 6 sm + 38 st-a	58	Pair 5 (t)*	Multiple: Pair 5 (t)*	Pair 1 (i)
							Pair 6 (t)	Pair 5 (t)*
*C. haroldoi*	Pavão river (PR)	**-**	48	6 m + 4 sm + 38 st-a	58	Pair 1 (i)	Simple: Pair1 (i)	Pair 1 (i)
*C.lepidota*	Barra do João Pedro (RS) and Miranda river (MS)	**-**	48	6 m + 42 st-a	54	Pair 1 (i)	Simple: Pair1 (i)	Pair 1 (i)
*C. punctata*	Saco da Alemoa and Forqueta river (RS)	**-**	48	4 m + 6 sm + 38 st-a	58	Pair 1 (i)	Simple: Pair 1 (i)	Pair 1 (i)
*C. maculata*	Maquiné river (RS)	**-**	48	6 m + 42 st-a	54	Pair 1 (i)*	Simple: Pair 1 (i)*	Pair 1 (i)*
*C. niederleinii*	Três Bocas stream (PR)	**-**	48	4 m + 6sm + 38st-a	58	Pair 1 (i)*	Simple: Pair 1(i)*	Pair 1 (i)*
*C. semifasciata*	Miranda river (MS)	**-**	48	4m+44st-a	52	Pair 1 (i)	Simpls: Pair 1 (i)	Pair 1 (i)

## Discussion

These are the first cytogenetic data for *C. haroldoi, C. maculata* and *C. punctata.* Along with data for *C. lepidota*, *C. niederleinii*, *C. semifasciata,* and *C. britskii,* all results presented a conserved diploid number (2n=48), corroborating data from the literature (Feldberg *et al.*, 2003; Benzaquem *et al.*, 2008). Thus far, all species of *Crenicichla* have shown this pattern, except *Crenicichla* sp studied by Rezende *et al.* (1996), which presented 2n=46. The FN is also consistent with the variations of 52 to 64 found in the literature (Pires, 2013). Despite the conservation of the diploid number, variations in the karyotype formulae were found in *C. semifasciata*, *C. niederleinii* and *C. britskii* in relation to other populations of these species (Feldberg and Bertollo, 1985a,b; Martins *et al.*, 1995; Benzaquem *et al.*, 2008; Poletto *et al.*, 2010). Such differences can be attributed to pericentric inversion events, which play an important role in the karyotype diversity of these species, as suggested by Feldberg and Bertollo (1985a).

According to Thompson (1979), the cichlids have 48 chromosomes of the subtelo-acrocentric type in basal species, where the presence of meta-submetacentric chromosomes would mean a derived karyotype. Furthermore, a greater presence of acrocentric chromosomes indicates a more ancestral karyotype. This hypothesis is shared by Feldberg *et al.* (2003), who consider the genus *Crenicichla* to be more derived because of the presence of meta- and submetacentric chromosomes. Considering this information, the genus *Crenicichla* is closer to Geophaginae, since the clade Cichlinae would be more ancestral because it presents mainly species with only subtelo-acrocentric chromosomes, as in the genus *Cichla* (Poletto *et al.*, 2010).

Another characteristic shared between the species analyzed, except for population A of *C. britskii,* was the presence of a secondary interstitial constriction on the first chromosome pair. This seems to be a chromosome characteristic of this genus and perhaps a cytotaxononomic marker, because it is also observed in *C. lacustris*, *C. semifasciata,* and *C. vittata* (Feldberg and Bertollo, 1985a,b), *C. lepidota* (Martins *et al.*, 1995; Perazzo *et al.*, 2011; Poletto *et al.*, 2010), *Crenicichla* sp., *C. niederleinii* (Loureiro *et al.*, 2000), *C. iguassuensis* (Mizoguchi *et al.*, 2007), and *C. reticulata* (Benzaquem *et al.*, 2008). This particular chromosome of the genus is another characteristic and makes this group similar to the clade Geohaginae, since other genera of this clade also present this type of chromosome, such as *Gymnogeophagus balzanii* (Feldberg and Bertollo, 1984; Roncati *et al.*, 2007), *Gymnogeophagus labiatus* (Pires *et al.*, 2010); *Geophagus surinamensis* (Feldberg and Bertollo, 1985a), and *Geophagus proximus* (Valente *et al.*, 2012).

Interestingly, population A of *C. britskii* did not show this constriction in the interstitial region but in the terminal region of the long arm of a submetacentric chromosome pair. Another interesting fact is that both populations of C. *britskii* presented a secondary constriction in the long arm in pair 20 (population A) and pair 5 (population B). The occurrence of these additional secondary constrictions has never been reported and may indicate a differential characteristic for this species.

The presence of a simple interstitial NOR in the first chromosome pair in all species, except *Crenicichla britskii,* and coincident with the secondary constriction, is well conserved in this genus, as reported by Loureiro *et al.* (2000), Roncati *et al.* (2007), Benzaquem *et al.* (2008) and Valente *et al.* (2012), among others. This trait varies only in the type of chromosomes, which may be metacentric (Martins *et al.*, 1995; Loureiro *et al.*, 2000; Mizoguchi *et al.*, 2007), or submetacentric (Martins *et al.*, 1995).

Occurrence of multiple NORs in population B of *C. britskii* may indicate that this population presents characteristics that are more derived in relation to the same species studied by Benzaquem *et al.* (2008) from another locality, which showed only a pair of NOR. This multiple pattern was previously reported in the genus, but only in *C. lepidota* from the region of Puerto Rico in the Paraná River basin (Martins *et al.*, 1995), which is a different situation from that found in *C. lepidota* in the present study.

All analyzed species of *Crenicichla,* except population B of *C. britskii,* showed only a pair of chromosomes with ribosomal cistron 18S, thus corroborating the data obtained by the impregnation of silver nitrate and the ancestral condition proposed by Feldberg *et al.* (2003). The hybridization signals were located interstitially on the short arm of the largest chromosome pair of the complement, similar to previously reported for *C. lepidota* (Perazzo *et al.*, 2010; Poletto *et al.*, 2010), the only species of the genus to date with results of *in situ* hybridization.

Size heteromorphism in the NORs, as found in pair 5 in *C. britskii* (population B), *C. niederleinii* and *C. maculata*, may be the result of irregular crossover or differential amplification of this region among the homologous chromosomes. This has previously been proposed for other fishes, including Cichlidae (Pires *et al.*, 2008; Gross *et al.*, 2010; Poletto *et al.*, 2010). The staining with CMA_3_ fluorochome evidenced fluorescent signals coincident with the NORs for the seven species, indicating the predominance of GC bases. However, population B of *C. britskii* again presented a distinct pattern with only one of the nucleolar pairs (pair 5) as CMA_3_ positive. NORs were negative for DAPI, thus revealing a scarcity of AT bases. The data with fluorochromes coincide with those reported for the genus by Loureiro *et al.* (2000), Perazzo *et al.* (2011), Mizoguchi *et al.* (2007), and Valente *et al.* (2012).

Among the species analyzed, *C. britskii* presented unique characteristics, despite having the same diploid number as the others members of the genus. The cytogenetic differences observed among the two populations of *C. britskii* may have resulted from geographic isolation between them. Ploeg (1991) also studied this species and found that it was endemic to the basin of Alto Paraná. This endemism resulted from the small displacement capacity of these fish: because they are highly territorial, they generally do not perform extensive migration throughout their life cycle and remain isolated (Castro, 1999).

According to Oliveira *et al.* (1988), populations that have less mobility and fewer individuals are more unstable in relation to their karyotype macrostructure. Gene flow is smaller, thus providing a higher rate of fixation of some chromosomal abnormality. This may be happening with the two populations of *C. britskii*, where geographic isolation would facilitate the establishment of chromosomal rearrangements and lead to a process of speciation. The population of *C*. *britskii* from the Paranapanema River has characteristics that are more derived when compared with the population from the Taquari Stream.

The results for the other species of *Crenicichla* show that karyotype patterns were similar to those found in the literature (Benzaquem *et al.*, 2008), indicating a conservative trend in chromosome evolution in this group of fish. However, the karyotype diversity found in populations of *C. britskii* provides new information about the karyotype evolution of the Cichlidae family. The cytogenetic characteristics that are particular to *Crenicichla* can be an important tool for phylogenetic studies in this group of fish, such as the largest pair of complement with secondary interstitial constriction and the presence of meta/sub metacentric chromosomes in the karyotype. This places the genus *Crenicichla* in the clade Geophaginae, which corroborates the phylogeny proposed by López-Fernández *et al.* (2005) and Smith *et al.* (2008).
